# Diffusing capacity as an independent predictor of acute exacerbations in chronic obstructive pulmonary disease

**DOI:** 10.1038/s41598-024-51593-8

**Published:** 2024-02-05

**Authors:** Heemoon Park, Hyo Jin Lee, Jung-Kyu Lee, Tae Yun Park, Kwang Nam Jin, Eun Young Heo, Deog Kyeom Kim, Hyun Woo Lee

**Affiliations:** 1 Division of Respiratory and Critical Care, Department of Internal Medicine, Seoul Metropolitan Government-Seoul National University Boramae Medical Center, Seoul, South Korea; 2 Department of Radiology, Seoul Metropolitan Government-Seoul National University Boramae Medical Center, Seoul, South Korea; 3 Department of Internal Medicine, Seoul National University College of Medicine, Seoul, South Korea; 4 Division of Pulmonary and Critical Care Medicine, Department of Internal Medicine, Seoul National University College of Medicine, Seoul Metropolitan Government-Seoul National University Boramae Medical Center, 20, Boramae-ro 5-gil, Dongjak-gu, Seoul, 07061 South Korea

**Keywords:** Diseases, Respiratory tract diseases, Chronic obstructive pulmonary disease

## Abstract

A weak correlation between diffusing capacity of the lung for carbon monoxide (DL_CO_) and emphysema has been reported. This study investigated whether impaired DL_CO_ in chronic obstructive pulmonary disease (COPD) is associated with increased risk of acute exacerbation independent of the presence or extent of emphysema. This retrospective cohort study included patients with COPD between January 2004 and December 2019. The participants were divided into four groups based on visually detected emphysema and impaired DL_CO_. Among 597 patients with COPD, 8.5% had no emphysema and impaired DL_CO_ whereas 36.3% had emphysema without impaired DL_CO_. Among the four groups, patients with impaired DL_CO_ and emphysema showed a higher risk of moderate-to-severe or severe exacerbation than those with normal DL_CO_. Impaired DL_CO_ was an independent risk factor for severe exacerbation (hazard ratio, 1.524 [95% confidence interval 1.121–2.072]), whereas the presence of emphysema was not. The risk of moderate-to-severe or severe exacerbation increases with the severity of impaired DL_CO_. After propensity-score matching for the extent of emphysema, impaired DL_CO_ was significantly associated with a higher risk of moderate-to-severe (*p* = 0.041) or severe exacerbation (*p* = 0.020). In patients with COPD and heterogeneous parenchymal abnormalities, DL_CO_ can be considered an independent biomarker of acute exacerbation.

## Introduction

The diffusion capacity of the lungs for carbon monoxide (DL_CO_) is a physiological indicator of parenchymal, alveolar, or capillary injury in chronic obstructive pulmonary disease (COPD). Impaired DL_CO_ is considered a poor prognostic factor in patients with COPD. Current smokers with impaired DL_CO_ had a higher risk of progression to COPD^1^. Impaired DL_CO_ is associated with worse respiratory symptoms, lower quality of life, decreased exercise performance, and a higher risk of severe exacerbation in COPD^2^. A prospective study reported that DL_CO_ was positively correlated with survival in patients with COPD^3^. Even in patients with mild COPD, DL_CO_ < 60% was a risk factor for all-cause mortality^4^. A previous meta-analysis showed that impaired DL_CO_ in COPD was associated with emphysema dominance and adverse clinical outcomes including exacerbation and mortality^5^.

Impaired DL_CO_ is believed to be primarily caused by emphysema in patients with COPD. The extent of emphysema is associated with the severity of DL_CO_ reduction^6–8^. Currently, the mechanism of how low diffusing capacity is related to poor prognosis in COPD patients has been explained by parenchymal destruction and loss of the pulmonary capillary bed due to emphysema^3,6^. As the amount of oxygen in the blood decreases with low DL_CO_, inflammatory mediators such as hypoxia-inducible factor are more likely to be expressed, which increases the risk of acute exacerbation (AE) of COPD^9,10^. However, the correlation coefficient between DL_CO_ and extent of emphysema was not sufficient to insist that emphysema is a major contributor to poor prognosis in patients with impaired DL_CO_^7,11^. Even in patients without emphysema, DL_CO_ may play an important role as a physiological indicator that reflects parenchymal, alveolar, or capillary injury and as a prognostic factor related to worse clinical outcomes in COPD.

Therefore, our study aimed to investigate whether impaired DL_CO_ in COPD patients is associated with increased risk of AE of COPD independent of emphysema.

## Materials and methods

This study was conducted in accordance with the Strengthening the Reporting of Observational Studies in Epidemiology statement^12^.

### Study design and participants

We analyzed patients who were diagnosed with COPD and followed up for 5 years in a teaching hospital between January 2004 and December 2019. Eligible patients had (1) post-bronchodilator forced expiratory volume in 1 s (FEV_1_)/forced vital capacity (FVC) < 0.7, with potential risk factors for COPD; (2) baseline and follow-up spirometric evaluation, including FEV_1_ and DL_CO_; and (3) baseline chest computed tomography (CT). The included patients were classified into four groups: no emphysema without impaired DL_CO_ (Group 1), no emphysema with impaired DL_CO_ (Group 2), emphysema without impaired DL_CO_ (Group 3), and emphysema with impaired DL_CO_ (Group 4). We excluded the patients who had asthma or severe anemia (hemoglobin < 8.0 g/dL).

This study was conducted according to the principles of the Declaration of Helsinki. The Institutional Review Board of Seoul Metropolitan Government-Seoul National University (SMG-SNU) Boramae Medical Center waived the requirement for written informed consent and approved this study (20-2022-80). It was confirmed that all procedures adhered to the relevant guidelines and regulations.

### Definition

Emphysema was defined as emphysema visually detected by an experienced radiologist (K.N.J.) on baseline chest CT. The extent of emphysema was evaluated using quantitative CT analysis of the percentage of lung voxels with attenuation of < − 950 Hounsfield units (%LAA-950). Impaired DL_CO_ was defined as DL_CO_ < 80%. We defined the severity of DL_CO_ as follows: normal, DL_CO_ ≥ 80%; mild, DL_CO_ ≥ 60% and < 80%; moderate, DL_CO_ ≥ 40% and < 60%; and severe, DL_CO_ < 40%^13^. Moderate exacerbation is defined as an increase in or new onset of respiratory symptoms requiring treatment with antibiotics and/or systemic steroid. Severe exacerbation is defined as an increase in or new onset of respiratory symptoms requiring hospitalization^14^.

### Variables

Baseline information including age, sex, body mass index (BMI), smoking history, Charlson comorbidity index (CCI), and respiratory morbidities was obtained. Clinical features including symptoms, previous history of exacerbation, Global Initiative for Chronic Obstructive Lung Disease (GOLD) group, blood test results, spirometric test results, radiologic findings, and inhaled treatments were collected.

### Outcomes

The study outcomes were moderate-to-severe and severe exacerbations in patients with COPD, classified according to emphysema and DL_CO_. Subgroup analyses were performed to evaluate the risk of moderate-to-severe or severe exacerbations according to DL_CO_ severity. For sensitivity analysis, a propensity score-matched analysis was performed to evaluate the risk of moderate-to-severe or severe exacerbation according to the severity of impaired DL_CO_.

### Statistical analyses

Analysis of variance or Kruskal–Wallis analysis was conducted to compare continuous variables. The chi-squared test or Fisher’s exact test was used to compare categorical variables. Cox regression analyses with backward elimination based on likelihood ratio tests were performed to identify clinical variables independently related to AE. The Kaplan–Meier (K–M) curve and log-rank test were used to compare the time to the first moderate-to-severe and severe exacerbation according to emphysema and the severity of impaired DL_CO_. For the sensitivity analysis, we performed 1:1 propensity score matching to evaluate the adjusted effect of DL_CO_ on AE. The propensity score was calculated using age, sex, BMI, smoking status, smoking amount (pack-years), CCI, moderate-to-severe exacerbation history, post-bronchodilator FEV_1_, and %LAA-950. A variance inflation factor > 4.0 was determined as significant multicollinearity. Statistical significance was set at two-tailed* p* < 0.05. R statistical software (version 4.1.2; R Foundation, Vienna, Austria) was used for statistical analyses.

### Ethics approval and consent to participate

This study was conducted according to the principles of the Declaration of Helsinki. The Institutional Review Board of Seoul Metropolitan Government-Seoul National University (SMG-SNU) Boramae Medical Center waived the requirement for written informed consent and approved this study (20-2022-80).

## Results

A total of 614 patients with COPD were followed for 5 years. After excluding 17 patients without DL_CO_ results or without baseline chest CT, the remaining 597 patients were divided into four groups based on visually detected emphysema and impaired DL_CO_ (Supplementary Fig. S1 online). In total, 115 (19.3%) patients had normal DL_CO_ without emphysema, 51 (8.5%) had impaired DL_CO_ without emphysema, 217 (36.3%) had normal DL_CO_ with emphysema, and 214 (35.8%) had impaired DL_CO_ with emphysema. Low correlations were found between %LAA-950 and emphysema (R^2^ = 0.144), %LAA-950 and DL_CO_ (R^2^ = 0.139), and emphysema and DL_CO_ (R^2^ = 0.024).

### Baseline characteristics and clinical features

Group 3 and 4 showed a higher age, more males, more ever-smokers, a higher pack-year, less history of asthma, less bronchiectasis, and higher %LAA-950 than group 1 and 2 (Table 1). Group 2 were younger, more likely to be female, and had less history of smoking than the other groups. In addition, group 2 stands out for a higher prevalence of tuberculosis and bronchiectasis compared to the other groups. There was significantly more sputum production (48.3% vs. 35.3%; *p* = 0.001) and dyspnea symptoms (COPD Assessment Test ≥ 10 or modified Medical Research Council score ≥ 2, 77.1% vs. 84.9%; *p* = 0.018) in group 2 and 4 than in group 1 and 3. Cough did not differ according to DL_CO_ or presence of emphysema (Table 2). Blood eosinophil counts did not differ among the four groups. The post-bronchodilator FEV_1_ was lower in group 2 and 4 than in group 1 and 3. Group 2 had lower post-bronchodilator FVC and higher post-bronchodilator FEV_1_/FVC than group 4. Regular inhalation treatment was used more frequently in group 2 and 4 than in group 1 and 3, whereas there was no difference in regular inhalation treatment when comparing group 1 and 2 to group 3 and 4.

**Table 1:** Baseline characteristics of COPD patients in unadjusted entire study population.

Variable	No emphysema without impaired DL_CO_ Group 1 (n = 115)	No emphysema with impaired DL_CO_ Group 2 (n = 51)	Emphysema without impaired DL_CO_ Group 3 (n = 217)	Emphysema with impaired DL_CO_ Group 4 (n = 214)	*p*-value
Age, year, mean (SD)	62.1 (10.9)	59.8 (13.3)	67.7 (10.0)	66.2 (9.4)	< 0.001
≥ 65, n (%)	55 (47.8)	20 (39.2)	141 (65.0)	122 (57.0)	0.001
≤ 50, n (%)	15 (13.0)	11 (21.6)	13 (6.0)	8 (3.7)	< 0.001
Male, n (%)	87 (79.8)	29 (61.7)	209 (97.7)	201 (95.3)	< 0.001
Body mass index, kg/m^2^, mean (SD)	23.1 (3.1)	22.4 (3.3)	22.5 (3.3)	21.6 (3.5)	0.001
Smoking status, n (%)					
Never smoker	37 (32.5)	21 (41.2)	12 (5.5)	12 (5.6)	< 0.001
Ex-smoker	43 (37.7)	15 (29.4)	107 (49.3)	106 (49.5)	
Current smoker	34 (29.8)	15 (29.4)	98 (45.2)	96 (44.9)	
Pack-years in ever smoker, mean (SD)	23.5 (23.9)	18.3 (21.3)	42.1 (26.0)	41.0 (25.4)	< 0.001
Comorbidities					
CCI, category, n (%)					
0–1	80 (69.6)	38 (74.5)	154 (71.0)	136 (63.6)	0.601
2–3	29 (25.2)	12 (23.5)	53 (24.4)	67 (31.3)	
≥ 4	6 (5.2)	1 (2.0)	10 (4.6)	11 (5.1)	
History of asthma, n (%)	38 (33.0)	17 (33.3)	63 (29.2)	44 (20.6)	0.043
History of tuberculosis, n (%)	28 (24.3)	24 (47.1)	43 (19.9)	64 (29.9)	0.001
Radiologic findings					
Bronchiectasis, n (%)	42 (36.5)	21 (41.2)	45 (20.7)	43 (20.1)	< 0.001
Interstitial lung disease, n (%)	3 (2.6)	0 (0.0)	2 (0.9)	4 (1.9)	0.564
%LAA-950, mean (SD)	2.6 (4.4)	4.0 (5.4)	7.4 (7.7)	14.0 (10.8)	< 0.001

Data are expressed as mean (± standard deviation) or number (percentage).

*CCI* Charlson comorbidity index, *COPD* chronic obstructive pulmonary disease, *DLco* diffusing capacity for carbon monoxide, *%LAA-950* percentage of lung voxels with attenuation <  − 950 Hounsfield units, *SD* standard deviation.

**Table 2:** Clinical features of COPD patients in unadjusted entire study population.

Variable	No emphysema without impaired DL_CO_ Group 1 (n = 115)	No emphysema with impaired DL_CO_ Group 2 (n = 51)	Emphysema without impaired DL_CO_ Group 3 (n = 217)	Emphysema with impaired DL_CO_ Group 4 (n = 214)	*p*-value
Symptoms and quality of life, n (%)					
Cough	8 (7.0)	8 (15.7)	14 (6.5)	19 (8.9)	0.172
Sputum	37 (32.2)	25 (49.0)	75 (34.7)	104 (48.6)	0.003
CAT ≥ 10 or mMRC ≥ 2	85 (78.0)	42 (89.4)	164 (76.6)	177 (83.9)	0.094
Previous exacerbation history, n (%)					
Moderate-to-severe	16 (14.7)	11 (23.4)	51 (23.8)	64 (30.3)	0.022
GOLD group, n (%)					
A	19 (17.4)	3 (6.4)	37 (17.3)	20 (9.5)	0.028
B	74 (67.9)	33 (70.2)	126 (58.9)	127 (60.2)	0.248
C	5 (4.6)	2 (4.3)	13 (6.1)	14 (6.6)	0.852
D	11 (10.1)	9 (19.1)	38 (17.8)	50 (23.7)	0.030
Blood test, mean (SD)					
White blood cell, /uL	7750 (2359)	8343 (3690)	7745 (2936)	8774 (3832)	0.009
Neutrophil, /uL	5035 (2208)	5529 (3568)	5012 (2839)	5963 (3794)	0.018
Lymphocyte, /uL	1971 (780)	1971 (920)	1956 (668)	2001 (970)	0.960
Neutrophil–lymphocyte ratio	3.22 (2.94)	3.96 (4.17)	3.06 (2.62)	4.14 (6.21)	0.077
Eosinophil, /uL	239 (240)	235 (324)	211 (195)	233 (286)	0.720
≥ 300, n (%)	29 (25.7)	12 (23.5)	45 (21.2)	55 (25.8)	0.691
Protein, g/dL	6.9 (0.6)	6.9 (067)	6.9 (0.5)	6.7 (0.6)	0.106
Albumin, g/dL	4.0 (0.4)	3.9 (0.5)	4.0 (0.4)	3.9 (0.4)	0.005
Spirometric test, mean (SD)					
Post-bronchodilator FEV_1_, L	1.82 (0.49)	1.31 (0.44)	1.80 (0.52)	1.50 (0.54)	< 0.001
Post-bronchodilator FEV_1_, %	71.78 (14.67)	56.44 (16.41)	72.23 (17.14)	59.29 (18.75)	< 0.001
Post-bronchodilator FVC, L	2.98 (0.78)	2.24 (0.76)	3.36 (0.70)	3.16 (0.85)	< 0.001
Post-bronchodilator FVC, %	83.64 (16.23)	67.75 (17.28)	93.51 (15.56)	86.49 (19.47)	< 0.001
Post-bronchodilator FEV_1_/FVC, %	61.03 (8.54)	60.12 (13.65)	53.10 (11.57)	47.79 (13.07)	< 0.001
Regular inhaled treatment, n (%)	100 (87.0)	48 (94.1)	179 (82.5)	199 (93.0)	0.004
LABA	2 (1.7)	2 (3.9)	4 (1.9)	7 (3.3)	0.596
LAMA	20 (17.4)	3 (5.9)	23 (10.6)	21 (9.8)	0.121
ICS/LABA	25 (21.7)	10 (19.6)	27 (12.5)	21 (9.8)	0.014
LABA/LAMA	40 (34.8)	18 (35.3)	84 (38.9)	75 (35.0)	0.824
ICS/LABA/LAMA	13 (11.3)	15 (29.4)	41 (19.0)	75 (35.0)	< 0.001
Use of ICS	38 (33.0)	25 (49.0)	68 (31.5)	96 (44.9)	0.007

Data are expressed as mean (± standard deviation) or number (percentage).

*CAT* COPD assessment test, *COPD* chronic obstructive pulmonary disease, *DLco* diffusing capacity for carbon monoxide, *FEV*_*1*_ forced expiratory volume in 1 s, *FVC* forced vital capacity, *GOLD* global initiative for chronic obstructive lung disease, *ICS* inhaled corticosteroid, *LABA* long-acting beta-agonist, *LAMA* long-acting muscarinic antagonist, *mMRC* modified medical research council, *SD* standard deviation.

### Moderate-to-severe exacerbation

**Figure 1:**
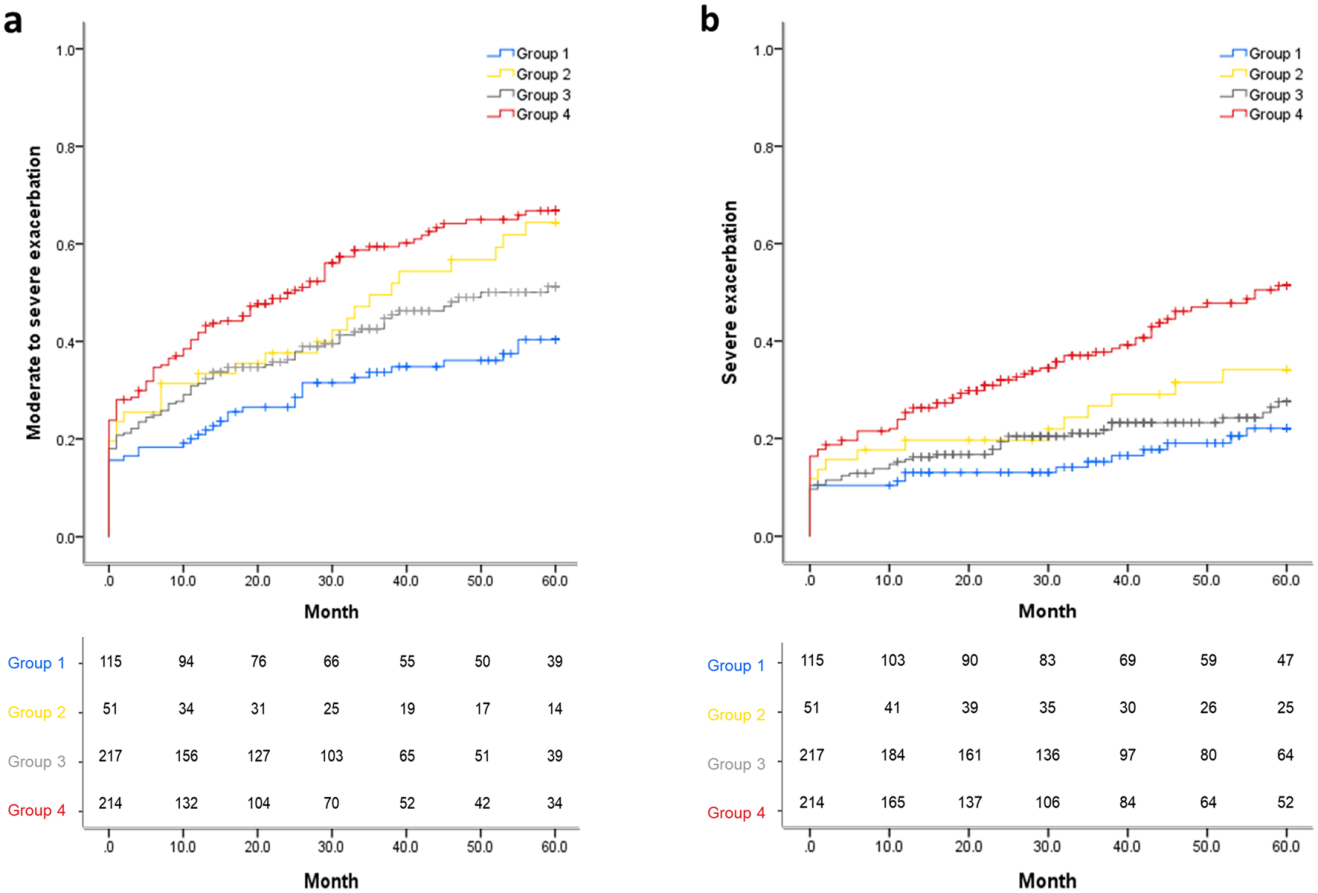
The time to the first (**a**) moderate to severe and (**b**) severe exacerbation analyzed by Kaplan–Meier curve and log-rank test according to emphysema and DL_CO_ in unadjusted entire study population. The included patients were classified into four groups: no emphysema without impaired DL_CO_ (Group 1), no emphysema with impaired DL_CO_ (Group 2), emphysema without impaired DL_CO_ (Group 3), and emphysema with impaired DL_CO_ (Group 4). Group 1 () vs. Group 2 (), Log-rank p-value = 0.012. Group 1 () vs. Group 3 (), Log-rank p-value = 0.058. Group 1 () vs. Group 4 (), Log-rank p-value < 0.001. Group 2 () vs. Group 3 (), Log-rank p-value = 0.250. Group 2 () vs. Group 4 (), Log-rank p-value = 0.375. Group 3 () vs. Group 4 (), Log-rank p-value = 0.002. Group 1 () vs. Group 2 (), Log-rank p-value = 0.109. Group 1 () vs. Group 3 (), Log-rank p-value = 0.270. Group 1 () vs. Group 4 (), Log-rank p-value < 0.001. Group 2 () vs. Group 3 (), Log-rank p-value = 0.392. Group 2 () vs. Group 4 (), Log-rank p-value = 0.055. Group 3 () vs. Group 4 (), Log-rank p-value < 0.001.

Moderate-to-severe exacerbation events occurred in 36.5% of group 1, 58.8% of group 2, 45.6% of group 3, and 60.3% of group 4. The time to the first moderate-to-severe exacerbation analyzed by K-M curve and log-rank test significantly differed among the four groups according to emphysema and DL_CO_ (log-rank *p* < 0.001; Fig. 1). Group 4 showed a higher risk of moderate-to-severe exacerbations than group 3 (log-rank *p* = 0.002). There was a significant difference in moderate-to-severe exacerbation between group 1 and 2 (log-rank *p* = 0.012). In the univariate Cox regression analysis, impaired DL_CO_ (*p* < 0.001) or emphysema (*p* = 0.013) was significantly associated with an increased risk of moderate-to-severe exacerbation (Table 3). However, this relationship disappeared in multivariate Cox regression analyses.

**Table 3:** Cox regression model for acute exacerbation of COPD patients in unadjusted entire study population.

Variable	Moderate-to-severe exacerbation				Severe exacerbation			
	Univariable		Multivariable		Univariable		Multivariable	
	Hazard ratio (95% CI)	*p*-value	Hazard ratio (95% CI)	*p*-value	Hazard ratio (95% CI)	*p*-value	Hazard ratio (95% CI)	*p*-value
Age	1.014 (1.004–1.025)	0.007			1.022 (0.008–1.036)	0.002	1.022 (1.005–1.039)	0.009
Male	0.902 (0.622–1.309)	0.588			0.923 (0.574–1.486	0.742		
Body mass index	0.938 (0.907–0.970)	< 0.001			0.905 (0.867–0.945)	< 0.001	0.956 (0.911–1.002)	0.063
Smoking status (ref.: never smoker)								
Ex-smoker	1.201 (0.852–1.691)	0.296			1.283 (0.811, 2.031)	0.287		
Current smoker	1.008 (0.708–1.434)	0.966			1.185 (0.742, 1.892)	0.478		
Charlson comorbidity index (ref.: 1)								
2–3	0.915 (0.706–1.184)	0.498			1.234 (0.896–1.700)	0.198	1.131 (0.816, 1.569)	0.459
≥ 4	1.270 (0.763–2.112)	0.357			2.048 (1.153–3.637)	0.014	2.120 (1.163–3.865)	0.014
History of asthma	1.227 (0.964–1.563)	0.096			1.191 (0.874–1.623)	0.268		
History of tuberculosis	1.183 (0.926–1.512)	0.178			1.142 (0.832–1.568)	0.412		
Previous moderate-to-severe exacerbation history	8.892 (6.858–11.528)	< 0.001	13.893 (7.361–26.221)	< 0.001	7.887 (5.814, 10.700)	< 0.001	6.577 (4.794–9.022)	< 0.001
GOLD group (Ref.: GOLD A)								
GOLD B	1.877 (1.119–3.148)	0.017	1.733 (0.955–3.144)	0.070	1.548 (0.743–3.229)	0.244		
GOLD C	11.655 (6.362–21.354)	< 0.001	0.722 (0.479–1.089)	0.120	14.117 (6.414–31.075)	< 0.001		
GOLD D	17.452 (10.120–30.095)	< 0.001			10.823 (5.222–22.432)	< 0.001		
Use of ICS	1.732 (1.384–2.168)	< 0.001			1.708 (1.261–2.313)	0.001		
Neutrophil–lymphocyte ratio	1.037 (1.022–1.053)	< 0.001			1.050 (1.035–1.064)	< 0.001		
Eosinophil ≥ 300/uL	1.159 (0.900–1.493)	0.254			0.881 (0.624, 1.242)	0.469		
Albumin, g/dL	0.495 (0.385–0.637)	< 0.001	0.634 (0.483–0.834)	0.001	0.331 (0.248–0.442)	< 0.001	0.461 (0.326–0.651)	< 0.001
Post-bronchodilator FEV_1_/FVC %	0.969 (0.960–0.979)	< 0.001	0.986 (0.976–0.996)	0.008	0.967 (0.955–0.979)	< 0.001		
Emphysema (ref. No emphysema)	1.399 (1.074–1.822)	0.013	0.989 (0.704–1.390)	0.951	1.662 (1.162–2.378)	0.005	1.017 (0.647–1.598)	0.941
With impaired DL_CO_ (ref. without impaired DLco)	1.585 (1.266–1.985)	< 0.001	1.116 (0.861–1.447)	0.407	2.032 (1.514–2.727)	< 0.001	1.524 (1.121–2.072)	0.007

*CAT* COPD assessment test, *CI* confidence interval, *COPD* chronic obstructive pulmonary disease, *CT* computed tomography, *DL*_*CO*_ diffusing capacity for carbon monoxide, *FEV*_*1*_ forced expiratory volume in 1 s, *FVC* forced vital capacity.

### Severe exacerbation

The time to first severe exacerbation analyzed by K–M curve and log-rank test was significantly different among the four groups (log-rank *p* < 0.001; Fig. 1). Group 4 showed a higher risk of severe exacerbation than group 1 and 3 (log-rank *p* < 0.001). In univariate Cox regression analysis, impaired DL_CO_ or emphysema was associated with a higher risk of severe exacerbation (Table 3). Even in multivariate Cox regression analysis, impaired DL_CO_ was associated with severe exacerbation (hazard ratio, 1.524; 95% confidence interval 1.121–2.072; *p* = 0.007).

### *Exacerbation and severity of impaired DL*_*CO*_

**Figure 2:**
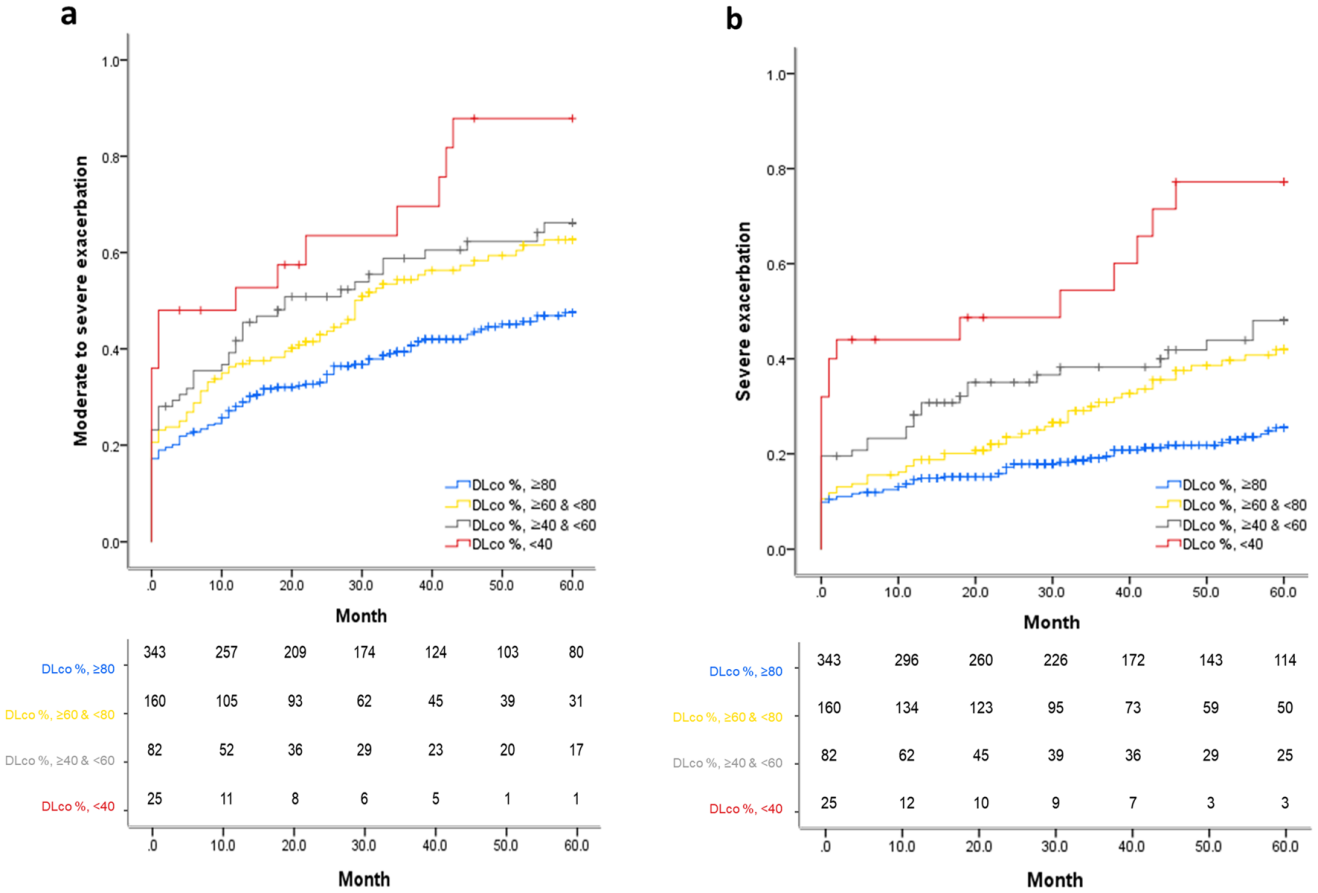
The time to the first (**a**) moderate-to-severe and (**b**) severe exacerbation analyzed by Kaplan–Meier curve and log-rank test according to DL_CO_ severity in unadjusted entire study population. DL_CO_ %, ≥ 80 () vs. DL_CO_ %, ≥ 60 & < 80 (), Log-rank p-value = 0.004. DL_CO_ %, ≥ 80 () vs. DL_CO_ %, ≥ 40 & < 60 (), Log-rank p-value = 0.002. DL_CO_ %, ≥ 80 () vs. DL_CO_ %, < 40 (), Log-rank p-value < 0.001. DL_CO_ %, ≥ 60 & < 80 () vs. DL_CO_ %, ≥ 40 & < 60 (), Log-rank p-value = 0.430. DL_CO_ %, ≥ 60 & < 80 () vs. DL_CO_ %, < 40 (), Log-rank p-value = 0.012. DL_CO_ %, ≥ 40 & < 60 () vs. DL_CO_ %, < 40 (), Log-rank p-value = 0.080. DL_CO_ %, ≥ 80 () vs. DL_CO_ %, ≥ 60 & < 80 (), Log-rank p-value = 0.002. DL_CO_ %, ≥ 80 () vs. DL_CO_ %, ≥ 40 & < 60 (), Log-rank p-value < 0.001. DL_CO_ %, ≥ 80 () vs. DL_CO_ %, < 40 (), Log-rank p-value < 0.001. DL_CO_ %, ≥ 60 & < 80 () vs. DL_CO_ %, ≥ 40 & < 60 (), Log-rank p-value = 0.207. DL_CO_ %, ≥ 60 & < 80 () vs. DL_CO_ %, < 40 (), Log-rank p-value < 0.001. DL_CO_ %, ≥ 40 & < 60 () vs. DL_CO_ %, < 40 (), Log-rank p-value = 0.011.

The time to the first moderate-to-severe or severe exacerbation analyzed by K-M curve and log-rank test was significantly different among the four groups according to the severity of impaired DL_CO_ (log-rank *p* < 0.001; Fig. 2). The risk of moderate-to-severe or severe exacerbation was significantly lower in patients with normal DL_CO_ than in those with any severity of impaired DL_CO_ (log-rank *p* < 0.005). In addition, the time to the first moderate-to-severe or severe exacerbation was significantly shorter in patients with severe DL_CO_ impairment than in those with mild or moderate impairment of DL_CO_ (log-rank *p* < 0.05) (Supplementary Fig. S2 online).

### Propensity score-matched analysis

**Figure 3:**
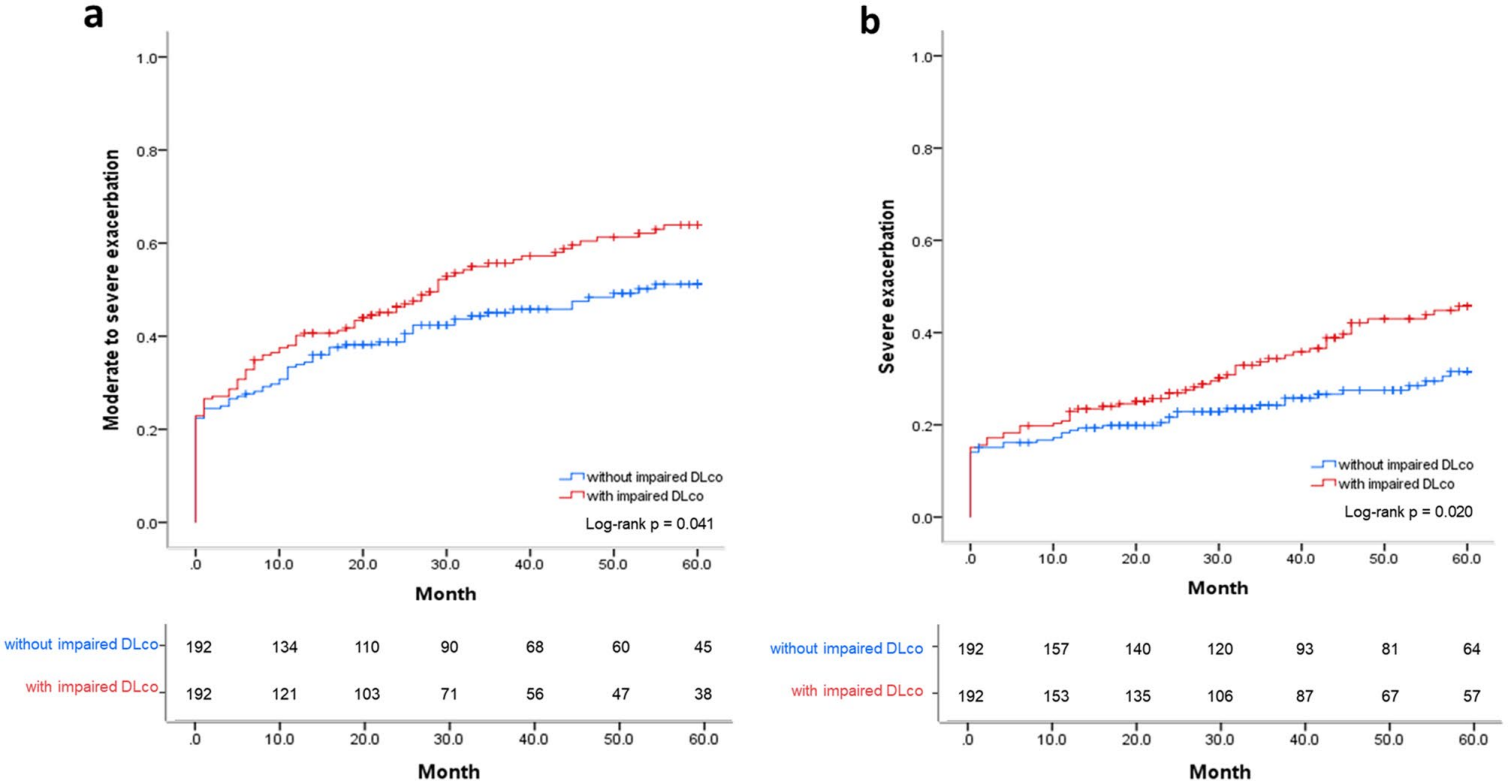
The time to the first (**a**) moderate to severe exacerbation or (**b**) severe exacerbation analyzed by Kaplan–Meier curve and log-rank test according to the group without impaired DL_CO_ and the group with impaired DL_CO_ in propensity score matched adjusted population. The propensity score was calculated using age, sex, BMI, smoking status, smoking amount (pack-years), CCI, moderate-to-severe exacerbation history, post-bronchodilator FEV_1_, and %LAA-950. *DL*_*CO*_ diffusing capacity for carbon monoxide, *FEV*_*1*_ forced expiratory volume in 1 s, *%LAA-950* percentage of lung voxels with attenuation < − 950 Hounsfield units.

After 1:1 propensity-score matching, the baseline severity of dyspnea, post-bronchodilator FEV_1_, previous moderate-to-severe exacerbation, and %LAA-950 were balanced between the groups with normal and impaired DL_CO_ (n = 192; Supplementary Tables S1 and S2 online). By using K-M curve and log-rank test, we found a significant difference in the time to the first moderate-to-severe (log-rank *p* = 0.041) and severe exacerbations (log-rank *p* = 0.020) between patients with normal and those with impaired DL_CO_ in the propensity score-matched population (Fig. 3).

## Discussion

Patients with COPD were classified into four groups based on visually detected emphysema and impairment of DL_CO_. Approximately half of patients with emphysema had normal DL_CO_, whereas 8.5% had impaired DL_CO_ without emphysema. Patients who had impaired DL_CO_ with emphysema showed a higher risk of moderate-to-severe or severe exacerbations than those with normal DL_CO_. In the multivariate analyses, impaired DL_CO_ was significantly associated with a higher risk of severe exacerbation, whereas the presence of emphysema was not. The risk of moderate-to-severe or severe exacerbation increases with the severity of impaired DL_CO_. In the propensity score-matched population, impaired DL_CO_ was significantly associated with a higher risk of moderate-to-severe or severe exacerbation. Therefore, DL_CO_ needs to be considered as a promising biomarker for the risk of future exacerbation in COPD patients with heterogeneous etiotypes.

Our study showed that impaired DL_CO_ was independently associated with moderate-to-severe or severe exacerbations, regardless of the presence of visually detected emphysema on chest CT. Impaired DL_CO_ is reportedly associated with an increased risk of AE in COPD. A previous study showed a significant association between impaired DL_CO_ (%) and severe exacerbation in multivariable analysis, which is consistent with our results^2^. Our study augmented existing knowledge by categorizing patients into four groups based on the presence of DL_CO_ impairment and emphysema. This categorization allowed us to contribute additional insights to the current understanding. By presenting the differences in baseline characteristics and clinical features among these groups, our study facilitated the development of plausible explanations for observed group differences. Through this stratification, we effectively excluded the potential correlation between DL_CO_ and emphysema. Subsequently, we presented HRs for AE in COPD using a Cox regression analysis. This approach is considered more robust for handling censoring data and provides an intuitive interpretation of the relationship between observed time and the occurrence of events. Furthermore, employing propensity score matching with clinical variables including the extent of emphysema (%LAA-950), our study revealed a significant difference in the time to AE between the normal and impaired DL_CO_ groups. These findings strongly suggest that impaired DL_CO_ may serve as a critical and independent risk factor for AE of COPD, regardless of the presence of emphysema.

The risk of AE was further increased when impaired DL_CO_ and chronic bronchitis were combined^15^. In a meta-analysis, a lower DL_CO_ was associated with a higher risk of exacerbation and mortality^5^. However, the mechanism by which impaired DL_CO_ is related to AE is not well identified. One plausible hypothesis is that DL_CO_ can accurately reflect the actual severity of emphysema and exercise tolerance^16,17^. Considering that low DL_CO_ is related with progression of airflow limitation even in healthy smokers with normal spirometric profiles, it is speculated that DL_CO_ can more sensitively detect the progression of small airway disease compared to other conventional spirometric parameters^1^. In addition, inflammatory mediators such as hypoxia-inducible factor are more likely to be expressed in hypoxemic conditions with impaired DL_CO_, which increases the risk of AE of COPD^9,10^. Based on our results, it could be assumed that impaired DLCO and emphysema have different mechanisms on AE of COPD.

Although emphysema is believed to be the main contributor to impaired DL_CO_ in patients with COPD, we found a discrepancy between visually detected emphysema and impaired DL_CO_ in 45% of patients. In addition, the correlation between emphysema and DL_CO_ is weak. Several studies have also reported a weak correlation between DL_CO_ and extent of emphysema. Among spirometric parameters, DL_CO_ had the highest correlation with the Visual Emphysema Score, but it was still a weak correlation (R^2^ = 0.438)^18^. The DL_CO_ corrected for alveolar volume (DL_CO_/VA) had a weak correlation with %LAA-950 (R^2^ = 0.417) and visual extent of emphysema (R^2^ = 0.411)^7^. DL_CO_/VA was better correlated with emphysema in COPD patients compared to healthy smokers, but the correlation between DL_CO_/VA and %LAA-950 is still suboptimal (R^2^ = 0.48)^19^. In fact, DL_CO_ can be impaired by bronchiectasis or tuberculosis-destroyed lung as well as emphysema. Impaired DL_CO_ was associated with an increasing number of bronchiectatic lobes^20^. The mean value of DL_CO_ in patients with pulmonary sequelae of tuberculosis was 74.1–78.8%^21,22^. Therefore, it would be better to understand the natural course of COPD with heterogeneous features by using DL_CO_ as a holistic index of parenchymal destruction rather than the extent of emphysema.

Patients with impaired DL_CO_ without emphysema tended to be younger, female, and had less of a smoking history compared to those with emphysema. Considering that they had a history of tuberculosis or bronchiectasis and a lower FVC, early life events of pneumonia or tuberculosis could be major contributing factors for the development of COPD. Therefore, impaired DL_CO_ without emphysema would be more likely found in young patients with COPD or COPD due to infections (COPD-I). The term “young COPD” has been suggested for those under 50 years of age with risk factors of COPD^23^. Young COPD is related with an increased risk of clinical COPD, hospitalization due to respiratory disease^24^, and mortality^25^. COPD-I is a currently proposed taxonomy for those with a history of early-life respiratory infection or tuberculosis. Especially in never-smokers, COPD-I is one of the major etiotypes of COPD^26,27^. Our study showed a significant difference in moderate-to-severe exacerbation between impaired DL_CO_ and normal DL_CO_ in patients without emphysema. Therefore, DL_CO_ may be a useful biomarker of AE in young patients with COPD or COPD-I.

This study has several limitations. First, our results cannot be generalized to a wider COPD population owing to its retrospective design. As we included patients from a single teaching hospital, there is likely selection bias, such as COPD patients with a higher symptom burden or more severe lung parenchymal destruction. Although we conducted a multivariate analysis and propensity-score matching, unmeasurable confounding variables may not have been sufficiently controlled. Second, emphysema was defined as emphysema visually detected by an experienced radiologist, which may have caused inter-observer variability. Although several studies have suggested the optimal cut-off of %LAA-950 to determine clinically relevant emphysema, we could not use it because various optimal cut-off values of %LAA-950 have been reported and other parenchymal abnormalities such as bronchiectasis or bulla can contribute to a larger %LAA-950. Third, DL_CO_ is affected by clinical factors beyond alveolar destruction, such as pulmonary vascular disease or obesity^28^. Indeed, DL_CO_ has been reportedly associated with pulmonary hypertension^29^. Considering the known association between pulmonary hypertension and an increased risk of severe exacerbations in patients with COPD, it is plausible that pulmonary hypertension may act as a mediating factor in the relationship between impaired DL_CO_ and an increased risk of exacerbation^30^. One of the limitations of the present study is the absence of an analysis on pulmonary hypertension.

## Conclusion

DL_CO_ may be an independent biomarker of AE in COPD patients with heterogeneous parenchymal abnormalities, regardless of the presence of emphysema.

### Supplementary Information

Supplementary Information 1.

Supplementary Information 2.

## Data Availability

The datasets used and/or analysed during the current study available from the corresponding author on reasonable request.
