# Supplementary material for: Diffusing capacity as an independent predictor of acute exacerbations in chronic obstructive pulmonary disease
Source: Sci Rep. 2024 Feb 5;14:2936. doi: 10.1038/s41598-024-51593-8 (PMC10844620; doi:10.1038/s41598-024-51593-8)
Supplement: Supplementary file 2 — Supplementary Information 2. [file 41598_2024_51593_MOESM2_ESM.docx]

**Diffusing Capacity as an Independent Predictor of Acute Exacerbations in Chronic Obstructive Pulmonary Disease**

**Authors:**

Heemoon Park M.D.^1^, Hyo Jin Lee, M.D.^1^, Jung-Kyu Lee, M.D.^1^, Tae Yun Park, M.D.^1^, Kwang Nam Jin, M.D.^3^, Eun Young Heo, Ph.D.^1^, Deog Kyeom Kim, M.D., Ph.D.^1,2^, *Hyun Woo Lee, M.D.^1^

**Affiliations:**

^1^Division of Respiratory and Critical Care, Department of Internal Medicine, Seoul Metropolitan Government-Seoul National University Boramae Medical Center, Seoul, South Korea

^2^Department of Internal Medicine, Seoul National University College of Medicine, Seoul, South Korea

^3^Department of Radiology, Seoul Metropolitan Government-Seoul National University Boramae Medical Center, Seoul, South Korea

**Corresponding author:**

*Hyun Woo Lee, M.D.

Division of Pulmonary and Critical Care Medicine, Department of Internal Medicine, Seoul Metropolitan Government-Seoul National University Boramae Medical Center, 20, Boramae-ro 5-gil, Dongjak-gu, Seoul, 07061, South Korea

Tel: +82-2-870-3432, Fax: +82-2-831-0714

E-mail: athrunzara86@snu.ac.kr

**Supplementary Figure Legends**

**Supplementary Fig. S1** Flow chart of the study participant selection

COPD, chronic obstructive pulmonary disease; DL_CO_, diffusing capacity of the lung for carbon monoxide; FEV_1_, forced expiratory volume in 1 second; FVC, forced vital capacity

**Supplementary Fig. S2** The time to the first (a) moderate-to severe and (b) severe exacerbation analyzed by Kaplan–Meier curve and log-rank test according to DL_CO_ severity (DL_CO_≥80%, ≥40% to <80%, <40%) in unadjusted entire study population

DL_CO_ %, ≥80 (**–**) vs. DL_CO_ %, ≥40 & <80 (**–**), Log-rank p-value<0.001

DL_CO_ %, ≥80 (**–**) vs. DL_CO_ %, <40 (**–**), Log-rank p-value<0.001

DL_CO_ %, ≥40 & <80 (**–**) vs. DL_CO_ %, <40 (**–**), Log-rank p-value=0.018

DL_CO_ %, ≥80 (**–**) vs. DL_CO_ %, ≥40 & <80 (**–**), Log-rank p-value<0.001

DL_CO_ %, ≥80 (**–**) vs. DL_CO_ %, <40 (**–**), Log-rank p-value<0.001

DL_CO_ %, ≥40 & <80 (**–**) vs. DL_CO_ %, <40 (**–**), Log-rank p-value<0.001

**Supplementary Table Legends**

**Supplementary Table S1** Baseline characteristics of the patients without impaired DL_CO_ and with impaired DL_CO_ before and after 1:1 propensity score matching

**Supplementary Table S2** Clinical features of the patients without impaired DL_CO_ and with impaired DL_CO_ before and after 1:1 propensity score matching

**Supplementary Fig. S1** Flow chart of the study participant selection


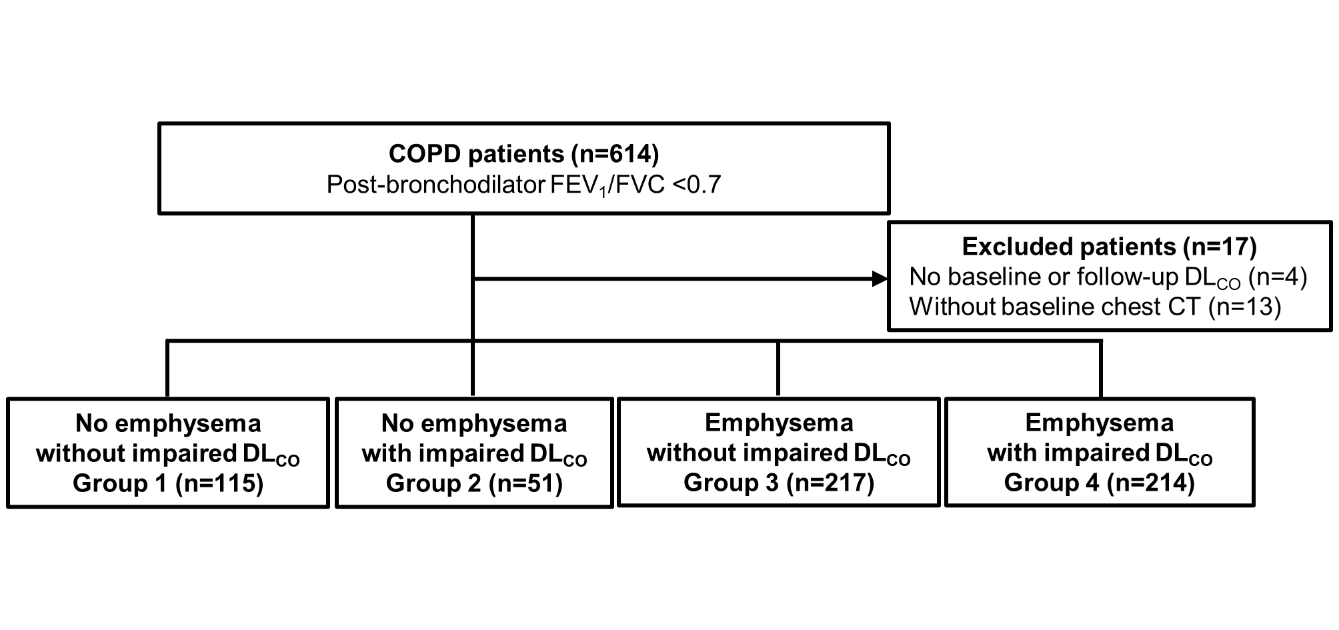


COPD, chronic obstructive pulmonary disease; DL_CO_, diffusing capacity of the lung for carbon monoxide; FEV_1_, forced expiratory volume in 1 second; FVC, forced vital capacity

**Supplementary Fig. S2** The time to the first (a) moderate-to severe and (b) severe exacerbation analyzed by Kaplan–Meier curve and log-rank test according to DL_CO_ severity (DL_CO_≥80%, ≥40% to <80%, <40%) in unadjusted entire study population

**
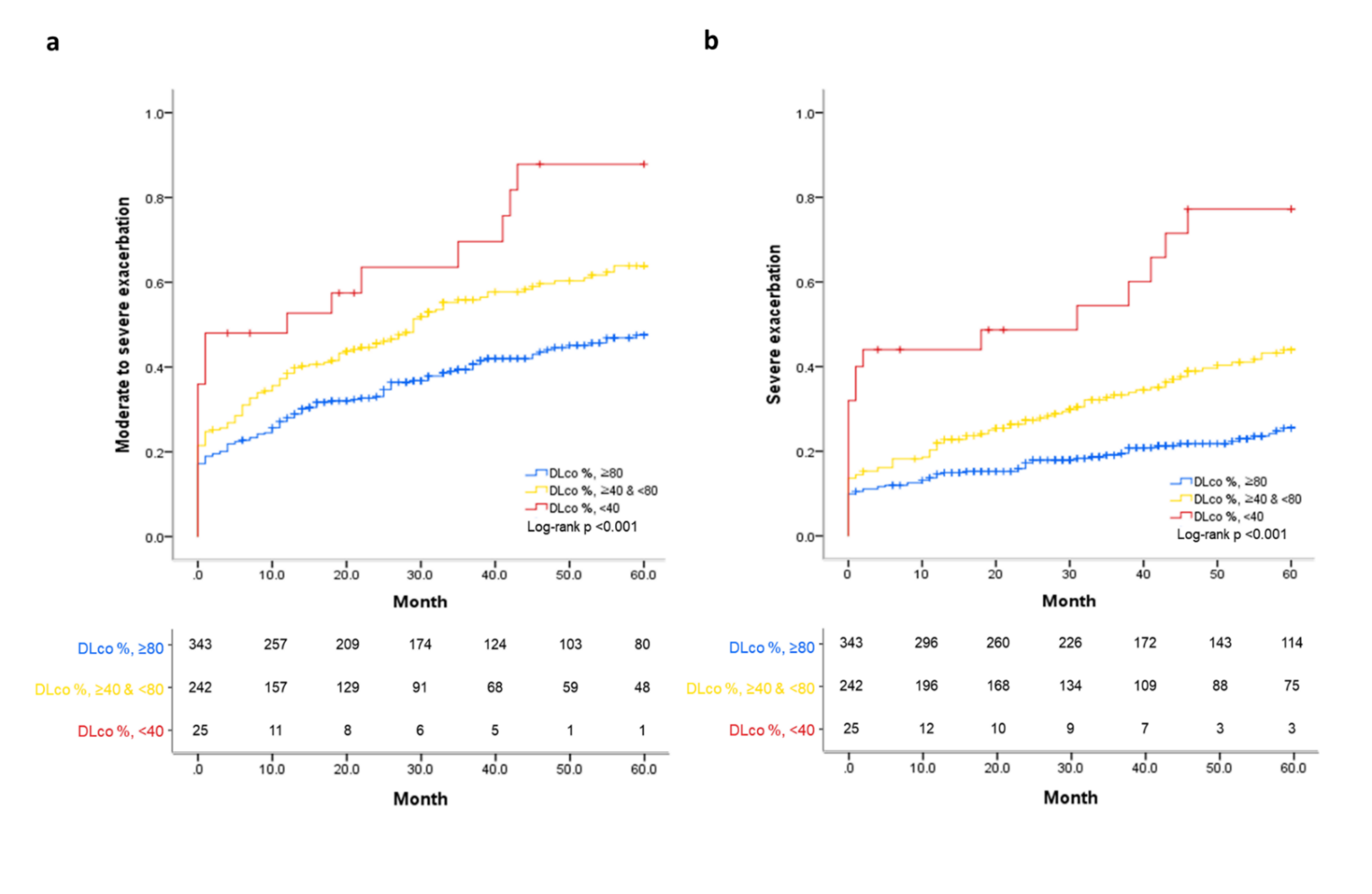
**

DL_CO_ %, ≥80 (**–**) vs. DL_CO_ %, ≥40 & <80 (**–**), Log-rank p-value<0.001 DL_CO_ %, ≥80 (**–**) vs. DL_CO_ %, ≥40 & <80 (**–**), Log-rank p-value<0.001

DL_CO_ %, ≥80 (**–**) vs. DL_CO_ %, <40 (**–**), Log-rank p-value<0.001 DL_CO_ %, ≥80 (**–**) vs. DL_CO_ %, <40 (**–**), Log-rank p-value<0.001

DL_CO_ %, ≥40 & <80 (**–**) vs. DL_CO_ %, <40 (**–**), Log-rank p-value=0.018 DL_CO_ %, ≥40 & <80 (**–**) vs. DL_CO_ %, <40 (**–**), Log-rank p-value<0.001

**Supplementary Table S1** Baseline characteristics of the patients without impaired DL_CO_ and with impaired DL_CO_ before and after 1:1 propensity score matching

| Variable | **Before PS matching** | | | **After PS matching** | | |
| --- | --- | --- | --- | --- | --- | --- |
|  | **with impaired DL_CO_ (n=267)** | **without impaired DL_CO_ (n=343)** | **SMD** | **with impaired DL_CO_ (n=192)** | **without impaired DL_CO_ (n=192)** | **SMD** |
| **Age, year, mean (SD)** | 64.8 (10.8) | 64.9 (11.7) | 0.009 | 65.5 (9.3) | 65.8 (9.7) | 0.032 |
| ≥65, n (%) | 142 (53.2) | 196 (57.1) | 0.088 | 101 (52.6) | 111 (57.8) | 0.116 |
| **Male, n (%)** | 230 (89.1) | 296 (91.6) | 0.007 | 174 (90.6) | 170 (88.5) | 0.124 |
| **Body mass index, kg/m^2^, mean (SD)** | 21.7 (3.4) | 22.8 (3.2) | 0.334 | 22.3 (3.4) | 22.5 (3.3) | 0.060 |
| **Smoking status, n (%)** |  |  |  |  |  |  |
| Never smoker | 33 (12.5) | 56 (16.4) | 0.176 | 25 (13.0) | 26 (13.5) | 0.025 |
| Ex-smoker | 121 (45.7) | 152 (44.4) | 0.027 | 85 (44.3) | 83 (43.2) | 0.023 |
| Current smoker | 111 (41.9) | 134 (39.2) | 0.062 | 82 (42.7) | 83 (43.2) | 0.012 |
| Pack-years in ever smoker, mean (SD) | 36.7 (26.2) | 34.6 (27.0) | 0.079 | 36.5 (26.3) | 37.6 (28.4) | 0.040 |
| **Comorbidities** |  |  |  |  |  |  |
| CCI, category, n (%) |  |  |  |  |  |  |
| 0-1 | 175 (65.5) | 244 (71.1) | 0.143 | 131 (68.2) | 133 (69.3) | 0.027 |
| 2-3 | 80 (30.0) | 83 (24.2) | 0.161 | 54 (28.1) | 52 (27.1) | 0.029 |
| ≥4 | 12 (4.5) | 16 (4.7) | 0.026 | 7 (3.6) | 7 (3.6) | 0 |
| History of asthma, n (%) | 61 (22.8) | 109 (31.9) | 0.250 | 47 (24.5) | 52 (27.2) | 0.079 |
| History of tuberculosis, n (%) | 90 (33.7) | 73 (21.3) | 0.348 | 63 (32.8) | 40 (20.9) | 0.337 |
| **Radiologic findings** |  |  |  |  |  |  |
| Bronchiectasis, n (%) | 64 (24.2) | 87 (26.2) | 0.041 | 47 (24.5) | 49 (25.5) | 0.031 |
| Interstitial lung disease, n (%) | 4 (1.5) | 5 (1.5) | 0.015 | 3 (1.6) | 2 (1.0) | 0.227 |
| %LAA-950, mean (SD) | 12.2 (10.7) | 5.8 (7.1) | 0.723 | 8.46 (7.65) | 7.68 (8.23) | 0.098 |

Data are expressed as mean (±standard deviation) or number (percentage).

PS, Propensity score; CCI, Charlson comorbidity index; COPD, chronic obstructive pulmonary disease; DL_CO_, diffusing capacity for carbon monoxide; %LAA-950, percentage of lung voxels with attenuation <-950 Hounsfield units; SD, standard deviation; SMD, standardized mean difference

Propensity score was calculated with age, sex, body mass index, smoking status, smoking amount (pack-years), Charlson comorbidity index, previous moderate-to-severe exacerbation history, post-bronchodilator FEV_1_ and %LAA-950.

**Supplementary Table S2** Clinical features of the patients without impaired DL_CO_ and with impaired DL_CO_ before and after 1:1 propensity score matching

|  | **Before PS matching** | | | **After PS matching** | | |
| --- | --- | --- | --- | --- | --- | --- |
|  | **with impaired DL_CO_ (n=267)** | **without impaired DL_CO_ (n=343)** | **SMD** | **with impaired DL_CO_ (n=192)** | **without impaired DL_CO_ (n=192)** | **SMD** |
| **Symptoms and quality of life, n (%)** |  |  |  |  |  |  |
| Cough | 27 (10.1) | 22 (6.4) | 0.273 | 19 (9.9) | 11 (5.8) | 0.323 |
| Sputum | 130 (48.7) | 113 (33.0) | 0.363 | 93 (48.4) | 72 (37.7) | 0.243 |
| CAT ≥10 or mMRC ≥2 | 219 (84.9) | 249 (77.1) | 0.300 | 161 (83.9) | 161 (83.9) | 0 |
| **Previous exacerbation history, n (%)** |  |  |  |  |  |  |
| Moderate-to-severe | 75 (29.1) | 67 (20.7) | 0.262 | 55 (28.6) | 48 (25.0) | 0.103 |
| **GOLD group, n (%)** |  |  |  |  |  |  |
| A | 23 (8.9) | 56 (17.3) | 0.420 | 17 (8.9) | 18 (9.4) | 0.035 |
| B | 160 (62.0) | 200 (61.9) | 0.002 | 120 (62.5) | 126 (65.6) | 0.075 |
| C | 16 (6.2) | 18 (5.6) | 0.063 | 14 (7.3) | 13 (6.8) | 0.044 |
| D | 59 (22.9) | 49 (15.2) | 0.279 | 41 (21.4) | 35 (18.2) | 0.109 |
| **Blood test, mean (SD)** |  |  |  |  |  |  |
| White blood cell, /uL | 8,686 (3,795) | 7,698 (2,748) | 0.304 | 9,035 (4,037) | 7,966 (2,710) | 0.311 |
| Neutrophil, /uL | 5,871 (3,744) | 4,978 (2,617) | 0.283 | 6,161 (4,019) | 5,218 (2,677) | 0.276 |
| Lymphocyte, /uL | 1,999 (959) | 1,957 (708) | 0.051 | 2,050 (999) | 1,968 (723) | 0.094 |
| Neutrophil-lymphocyte ratio | 4.10 (5.85) | 3.09 (2.70) | 0.231 | 4.30 (6.63) | 3.25 (2.73) | 0.207 |
| Eosinophil, /uL | 233 (293) | 224 (217) | 0.036 | 231 (306) | 223 (224) | 0.030 |
| ≥300, n (%) | 67 (25.3) | 78 (23.3) | 0.071 | 47 (24.6) | 45 (23.8) | 0.024 |
| Protein, g/dL | 6.8 (0.6) | 6.9 (0.6) | 0.167 | 6.8 (0.7) | 6.9 (0.6) | 0.153 |
| Albumin, g/dL | 3.9 (0.4) | 4.0 (0.4) | 0.250 | 3.9 (0.4) | 4.0 (0.4) | 0.250 |
| **Spirometric test, mean (SD)** |  |  |  |  |  |  |
| Post-bronchodilator FEV_1_, L | 1.45 (0.54) | 1.85 (0.56) | 0.726 | 1.58 (0.52) | 1.62 (0.46) | 0.082 |
| Post-bronchodilator FEV_1_, % | 57.47 (18.55) | 71.75 (16.70) | 0.814 | 62.55 (17.05) | 64.48 (14.43) | 0.122 |
| Post-bronchodilator FVC, L | 3.04 (0.90) | 3.33 (0.78) | 0.348 | 3.11 (0.90) | 3.16 (0.72) | 0.061 |
| Post-bronchodilator FVC, % | 83.73 (19.86) | 91.22 (15.73) | 0.423 | 85.21 (18.95) | 88.58 (15.5) | 0.195 |
| Post-bronchodilator FEV_1_/FVC, % | 48.06 (11.71) | 54.94 (10.32) | 0.628 | 51.48 (10.45) | 51.33 (10.82) | 0.014 |
| **Inhaled treatment, n (%)** |  |  |  |  |  |  |
| No regular inhaled treatment | 18 (6.7) | 52 (15.2) | 0.501 | 16 (8.3) | 21 (10.9) | 0.166 |
| LABA | 9 (3.4) | 6 (1.8) | 0.369 | 8 (4.2) | 2 (1.0) | 0.779 |
| LAMA | 25 (9.4) | 47 (13.7) | 0.239 | 18 (9.4) | 18 (9.4) | 0 |
| ICS/LABA | 32 (12.0) | 58 (17.0) | 0.224 | 26 (13.5) | 29 (15.2) | 0.074 |
| LABA/LAMA | 93 (34.8) | 125 (36.5) | 0.041 | 70 (36.5) | 84 (44.0) | 0.173 |
| ICS/LABA/LAMA | 90 (33.7) | 54 (15.8) | 0.550 | 54 (28.1) | 38 (19.9) | 0.251 |
| Use of ICS | 122 (45.7) | 112 (32.7) | 0.302 | 80 (41.7) | 67 (35.1) | 0.154 |

Data are expressed as mean (±standard deviation) or number (percentage). Propensity score was calculated with age, sex, body mass index, smoking status, smoking amount (pack-years), Charlson comorbidity index, previous moderate-to-severe exacerbation history, post-bronchodilator FEV_1_ and %LAA-950.

PS, Propensity score; CAT, COPD assessment test; COPD, chronic obstructive pulmonary disease; DL_CO_, diffusing capacity for carbon monoxide; FEV_1_, forced expiratory volume in 1 second; FVC, forced vital capacity; GOLD, Global Initiative for Chronic Obstructive Lung Disease; ICS, inhaled corticosteroid; LABA, long-acting beta-agonist; LAMA, long-acting muscarinic antagonist; mMRC, modified medical research council; SD, standard deviation; %LAA-950, percentage of lung voxels with attenuation <-950 Hounsfield units; SMD, standardized mean difference
